# The Comparison between Robotic and Manual Ablations in the Treatment of Atrial Fibrillation: A Systematic Review and Meta-Analysis

**DOI:** 10.1371/journal.pone.0096331

**Published:** 2014-05-06

**Authors:** Wenli Zhang, Nan Jia, Jinzi Su, Jinxiu Lin, Feng Peng, Wenquan Niu

**Affiliations:** 1 Department of Cardiology, Fuzhou General Hospital of Nanjing Command, PLA, Fujian Medical University, Fuzhou, Fujian, China; 2 Department of Cardiology, The Fourth People's Hospital of Shenzhen, Shenzhen, Guangdong, China; 3 Department of Cardiology, The First Affiliated Hospital of Fujian Medical University, Fuzhou, Fujian, China; 4 State Key Laboratory of Medical Genomics, Ruijin Hospital, Shanghai Jiao Tong University School of Medicine, Shanghai, China; 5 Shanghai Institute of Hypertension, Ruijin Hospital, Shanghai Jiao Tong University School of Medicine, Shanghai, China; Johns Hopkins University SOM, United States of America

## Abstract

**Objective:**

To examine in what aspects and to what extent robotic ablation is superior over manual ablation, we sought to design a meta-analysis to compare clinical outcomes between the two ablations in the treatment of atrial fibrillation.

**Methods and Results:**

A literature search was conducted of PubMed and EMBASE databases before December 1, 2013. Data were extracted independently and in duplicate from 8 clinical articles and 792 patients. Effect estimates were expressed as weighted mean difference (WMD) or odds ratio (OR) and the accompanied 95% confidence interval (95% CI). Pooling the results of all qualified trials found significant reductions in fluoroscopic time (minutes) (WMD; 95% CI; P: -8.9; -12.54 to -5.26; <0.0005) and dose-area product (Gy×cm^2^) (WMD; 95% CI; P: -1065.66; -1714.36 to -416.96; 0.001) for robotic ablation relative to manual ablation, with evident heterogeneity (P<0.0005) and a low probability of publication bias. In subgroup analysis, great improvement of fluoroscopic time in patients with robotic ablation was consistently presented in both randomized and nonrandomized clinical trials, particularly in the former (WMD; 95% CI; P: -12.61; -15.13 to -10.09; <0.0005). Success rate of catheter ablation was relatively higher in patients with robotic ablation than with manual ablation (OR; 95% CI; P: 3.45; 0.24 to 49.0; 0.36), the difference yet exhibiting no statistical significance.

**Conclusions:**

This study confirmed and extended previous observations by quantifying great reductions of fluoroscopic time and dose-area product in patients referred for robotic ablation than for manual ablation in the treatment of atrial fibrillation, especially in randomized clinical trials.

## Introduction

Treating atrial fibrillation via catheter ablation has long been established as a safe and effective strategy [1]. Recent years have witnessed extraordinary innovation in catheter ablation from conventional manual approach to the robotic-guided navigation system [2], [3]. Nonetheless, the benefits of robotic ablation over manual ablation are currently subject to an ongoing debate [4]. For example, in a relatively large clinical trial by Thomas et al [5], early use of robotic ablation led to a significant reduction of fluoroscopic time compared with manual ablation, whereas there was no material difference between the two ablations in another clinical trial by Rillig et al [6]. However, it should be noted that the majority of these clinical trials have been seriously underpowered, and some are even nonrandomized. In this context, a meta-analysis represents a powerful statistical methodology for synthesizing research evidence across independent trials [7]. Given the accumulation of data, we sought to design a meta-analysis to compare procedure outcomes between robotic and manual ablations in terms of fluoroscopic time, total procedure duration, radiofrequency time and dose-area product, as well as the success rates of catheter ablation and its major complications in the treatment of atrial fibrillation.

## Methods

We undertook this meta-analysis of clinical trials in conformity with the guidelines put forth by the Preferred Reporting Items for Systematic Reviews and Meta-analyses (PRISMA) statement (Checklist S1) [8].

### Search Strategy

A literature search was conducted of PubMed and EMBASE databases covering the period from the earliest possible year to December 1, 2013, with search terms including “ablation”, “robotic”, or “navigation”, annexed with “atrial fibrillation” or “arrhythmias”. In addition, this search was complemented with the perusal of the bibliographies of retrieved original reports and review articles to identify additional eligible articles. Search results were restricted to English-language and clinical trials.

### Trial Selection

Two investigators (F.P. and W.N.) independently read the titles and abstracts to assess their eligibility, and they retrieved the full texts of potentially eligible articles. When necessary, we emailed the corresponding authors to avoid the double counting of study groups involved in more than one clinical trial. Where more than one publication of a clinical trial existed, we extracted data from the most recent or complete publication.

### Eligibility Criteria

For inclusion, trials had to involve patients needing catheter ablation treatment for atrial fibrillation and compare the changes of either fluoroscopic time, total procedure time, radiofrequency time or dose-area product between robotic and manual ablations. Trials were excluded if they were cross-over trials or conference abstracts or proceedings, case reports or series, editorials, narrative reviews, or non-English articles.

### Data Extraction

Two investigators (F.P. and W.N.) independently extracted data using a standardized Excel template (Microsoft Corp, Redmond, WA). Disagreements were settled by consensus.

For each article, the following data were summarized: the first author's surname, year of publication, ethnicity of study patients, sample size of each treatment, fluoroscopic time (minutes), total procedure time (minutes), radiofrequency time (minutes), dose-area product (Gy×cm^2^), the success rate of catheter ablation, and major complication rate between two ablations, as well as the characteristics of trial patients including age, gender, body mass index, atrial fibrillation duration (years), left atrium size (mm), the percentages of paroxysmal atrial fibrillation, left ventricular ejection fraction (LVEF), coronary artery disease (CAD), hypertension and diabetes.

Total procedure time was defined as the time from venous puncture until sheath withdrawal. Radiofrequency time, also known as ablation time, was defined as the time from the first to the last ablation. The success of catheter ablation was defined as complete pulmonary vein isolation, which was confirmed by the disappearance of all pulmonary vein potentials or the dissociation of pulmonary vein potentials from left atrial activity. Major complications referred to adverse events causing either temporary or permanent change in health status requiring intervention.

Diagnosis of hypertension was based on the presence of elevated systolic (≥140 mmHg) and/or diastolic (≥90 mmHg) blood pressure, or current use of antihypertensive medications. Diabetes was defined as fasting plasma glucose levels ≥7.0 mmol/L or non-fasting plasma glucose levels ≥11.0 mmol/L, or taking hypoglycemic drugs or receiving parenteral insulin therapy.

### Statistical Analysis

For a certain clinical outcome, where data from three or more independent trials were available, a meta-analysis was done. Quantitative outcomes were summarized and compared by weighted mean difference (WMD) with 95% confidence interval (95% CI) between robotic and manual ablations. Categorical variables were compared between the two groups by weighted odds ratio (OR) and its corresponding 95% CI. For each study, weight was calculated as the reciprocal of the variance of the estimated intervention effect. The random-effects model using the DerSimonian & Laird method [9] was employed irrespective of the existence of heterogeneity. Heterogeneity across studies was examined with the inconsistency index (*I* ^2^) test, which ranges from 0 to 100% and is defined as the percentage of the observed variability that is due to heterogeneity rather than chance. Given the limited power of *I* ^2^ test for a small number of studies, we considered the presence of heterogeneity at 10% level of significance.

Predefined subgroup analysis was conducted a priori according to study design (randomized and nonrandomized clinical trials). Sensitivity analysis was performed to assess the contribution of individual trials to pooled effect estimates by sequentially omitting each trial one at a time and computing differential estimates for remaining trials. Meta-regression analysis was carried out to evaluate the extent to which different trial-level variables including all characteristics of trial patients as mentioned above explained the heterogeneity of different effect estimates between robotic and manual ablations.

Begg's funnel plot was constructed for assessment of publication bias. The asymmetry of this plot was assessed by Egger's regression test and then corrected by the trim and fill method with the adjusted effect estimates and number of studies. Also considering the small number of studies involved in this meta-analysis, we considered the presence of publication bias at 10% level of significance for Egger's regression test [10]. Statistical analyses were completed with the use of STATA software (StataCorp, College Station, TX, version 11.2 for Windows).

## Results

### Eligible Trials

**Figure 1:**
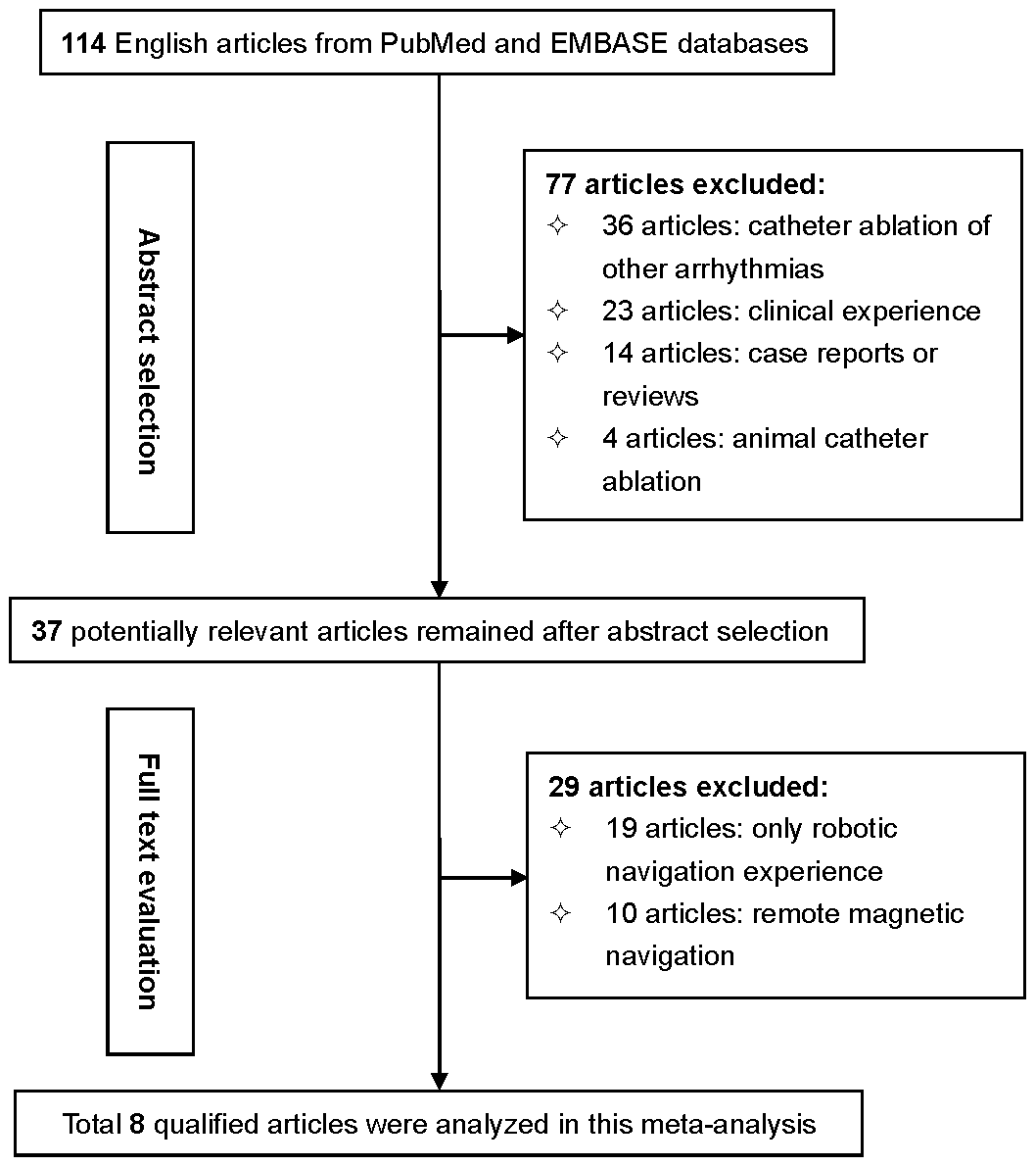
Flow diagram of search strategy and study selection.

The characteristics of study patients involved in all qualified trials are summarized in Table 1 and Table 2. The primary search for clinical trials comparing the procedure outcomes between robotic and manual ablations yielded 114 potentially relevant articles published in English language. Figure 1 illustrates a flow diagram schematizing the process of excluding articles with specific reasons. Consequently, 8 articles met our selection criteria and were published from the year 2009 to 2013 [2], [5], [6], [11]-[15]. Two of 8 qualified articles recorded outcomes with more than one bipolar voltage of radiofrequency ablation [11], [13], resulting in a total of 10 trials in the final analysis.

**Table 1:** Baseline characteristics of study patients in qualified studies.

Author (year)	Country	Random	Number	Age (year)	Gender (Male)	AF duration (year)	BMI (kg/m^2^)	LA size (mm)
Malcolme-Lawes et al (2013) (30 W)	UK	Yes	10/10	59.3/64.6	NA	3.28/3.48	NA	42.7/38
Malcolme-Lawes et al (2013) (60 W)	UK	Yes	10/10	60.6/64.6	NA	4.99/3.48	NA	42.5/38
Thomas et al (2012)	Germany	No	25/61	60/62	0.64/0.8	4.9/5.5	NA	41/42
Rillig et al (2012)	Germany	No	50/20	60/66.5	0.58/0.75	NA	26.7/26.9	47.5/47.5
Duncan et al (2012)	UK	Yes	21/23	53/55	0.62/0.61	6.83/6.67	NA	NA
Tilz et al (2010) (20 W)	Germany	No	10/25	58/61	0.6/0.56	8.3/8.5	26/25	44/44
Tilz et al (2010) (30 W)	Germany	No	4/25	59/61	0.75/0.56	13/8.5	28/25	48/44
Steven et al (2010)	Germany	Yes	30/30	62/61	0.67/0.47	7/6	NA	40/39
Kautzner et al (2009)	Czech	No	22/16	55/55	0.73/0.81	NA	NA	NA
Di Biase et al (2009)	USA	No	193/197	63/61	0.75/0.74	4.25/4.25	30/30	42.3/44

**Table 2:** Baseline characteristics of study patients in qualified studies.

Paroxysmal AF (%)	LVEF (%)	CAD (%)	Hypertension (%)	Diabetes (%)	Freedom from AF (%)	Major complications (%)
100/100	56.2/52	9.1/11.1	27.3/55.6	0/22.2	50/60	10/0
100/100	56.4/52	11.1/11.1	33.3/55.6	11.1/22.2	80/60	0/0
76/52	NA	28/16	72/62	NA	NA	4/5
58/60	NA	14/5	62/75	8/10	NA	NA
100/100	NA	NA	NA	NA	NA	NA
NA	NA	NA	60/72	NA	NA	NA
NA	NA	NA	75/72	NA	NA	NA
100/100	68/67	13/7	73/80	NA	73/77	NA
100/100	NA	NA	NA	NA	NA	NA
66/69	58/57	22/21	65/50	8/9	100/68	2/1

*Abbreviations*: AF, atrial fibrillation; BMI, body mass index; LA, left atrium; LVEF, left ventricular ejection fraction; CAD, coronary artery disease; NA, not available.

Data are expressed as mean values or percentages unless otherwise indicated between robotic and manual ablations.

All 10 qualified trials were conducted in Caucasian populations, and four of them were on a randomized design [2], [11], [12]. There were respectively total 375 and 417 patients assigned to the robotic ablation and manual ablation procedures in atrial fibrillation treatment. Patients with robotic ablation (mean age: 59.0 years) were younger than those with manual ablation (61.2 years, P = 0.03), and there were no distribution differences for gender and body mass index between the two procedures. The average values of atrial fibrillation duration (6.57 years versus 5.8 years) and left atrium size (43.5 mm versus 42.06 mm) were slightly larger in patients with robotic ablation than with manual ablation group. The percentages of paroxysmal atrial fibrillation, LVEF, CAD, hypertension and diabetes were comparable between the two procedures.

### Overall and Subgroup Analyses

**Figure 2:**
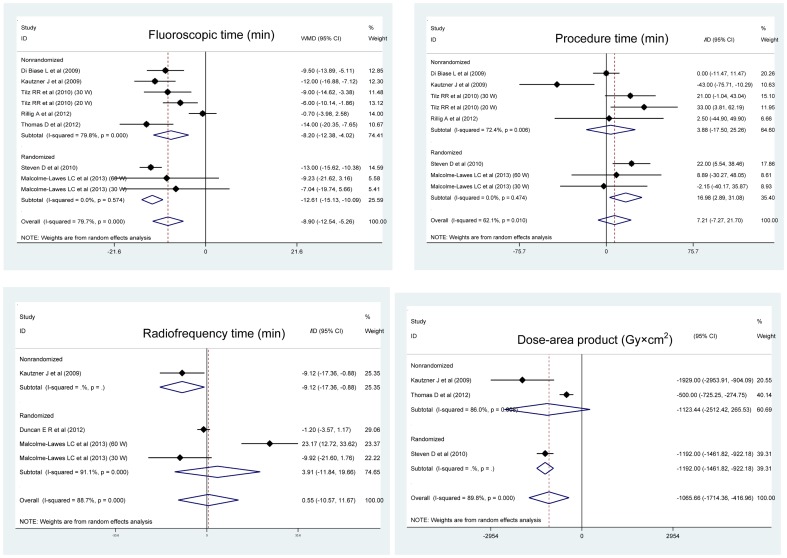
Forest plots of changes of fluoroscopic time, total procedure time, radiofrequency time, dose-area product for the comparison of robotic ablation with manual ablation.

**Figure 3:**
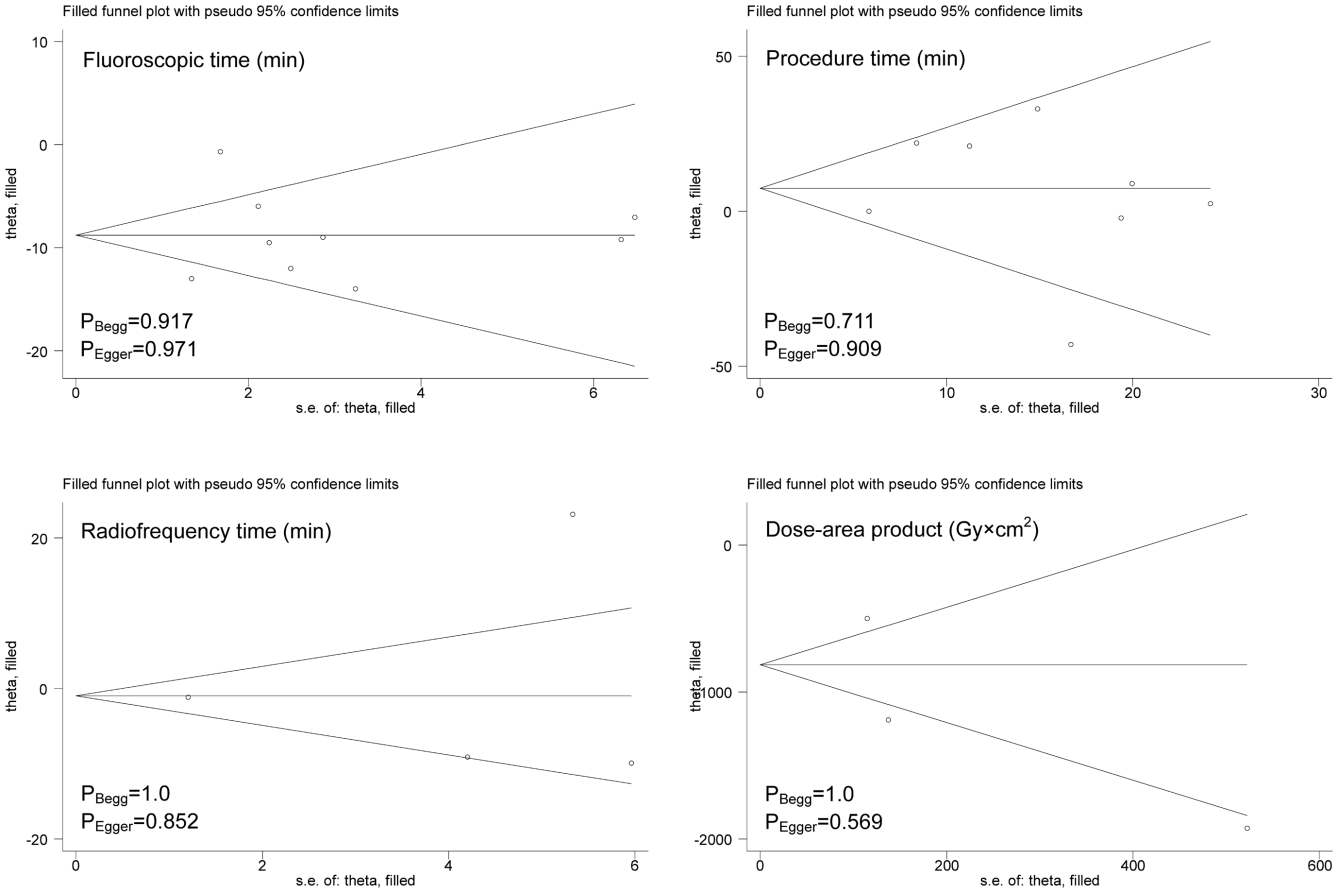
Filled funnel plots of fluoroscopic time, total procedure time, radiofrequency time, dose-area product for the comparison of robotic ablation with manual ablation.

Overall effect estimates for fluoroscopic time, total procedure time, radiofrequency time, and dose-area product, as well as the corresponding subgroup analyses by study design are presented in Figure 2. Pooling the results of all qualified trials observed significant reductions in fluoroscopic time (minutes) (WMD; 95% CI; P: -8.9; -12.54 to -5.26; <0.0005) and dose-area product (Gy×cm^2^) (-1065.66; -1714.36 to -416.96; 0.001) for robotic ablation compared with manual ablation, accompanying strong evidence of heterogeneity for both estimates (P<0.0005) and low probability of publication bias as estimated by the Begg's and Egger's tests and the visual inspection of funnel plots based on the trim and fill method (Figure 3). The differences in the magnitude of total procedure time and radiofrequency time were matched between robotic and manual ablations with the presence of heterogeneity (Figure 2) and the absence of publication bias (Figure 3).

In subgroup analyses, great improvement of fluoroscopic time in patients with robotic ablation was consistently seen in both randomized and nonrandomized clinical trials, particularly in the former (WMD; 95% CI; P: -12.61; -15.13 to -10.09; <0.0005). Moreover, there was no indication of heterogeneity (I^2^ = 0.0%; P = 0.574) and publication bias (data not shown) for randomized clinical trials. As for dose-area product, significant difference was only noticed in randomized trials (-1192.0; -1461.82 to -922.18), which involved only one trial.

### Success Rates and Major Complications

**Figure 4:**
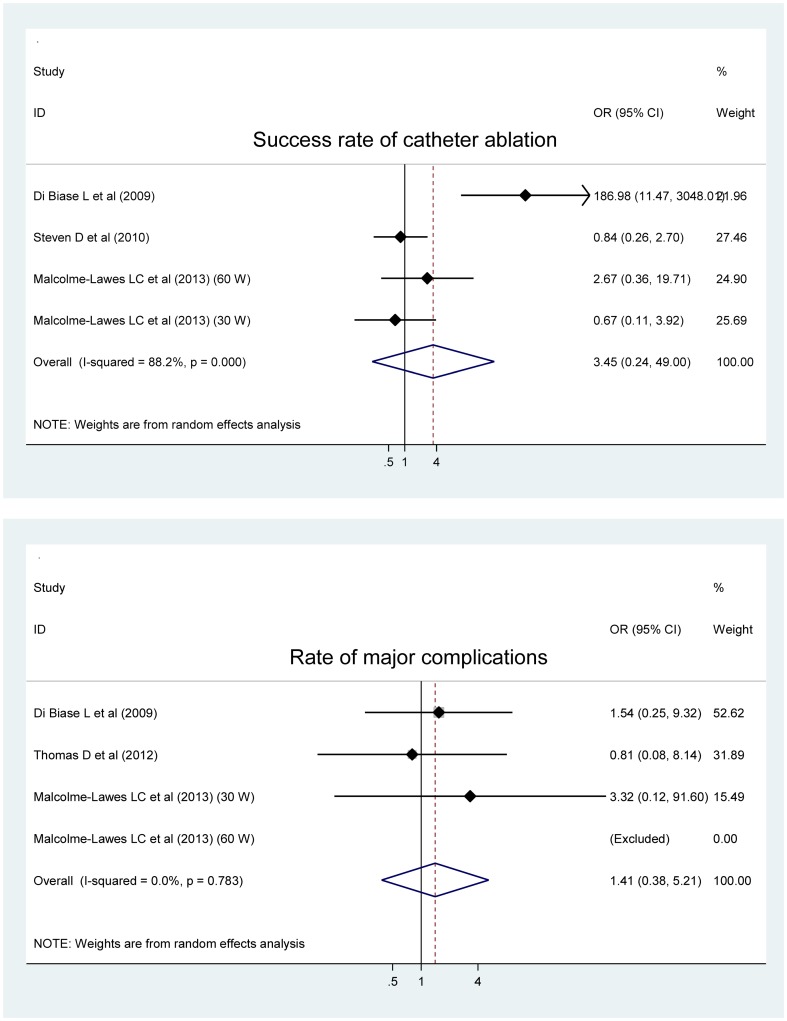
Forest plots of changes of the successful procedure of catheter ablation and the incidence of major complications for the comparison of robotic ablation with manual ablation.

Success rate of catheter ablation was relatively higher in patients with robotic ablation than with manual ablation (OR; 95% CI; P: 3.45; 0.24 to 49.0; 0.36), the difference yet exhibiting no statistical significance (Figure 4). Similarly for major complications, robotic ablation approach was associated with slightly high rate compared with manual ablation approach (1.41; 0.38 to 5.21; 0.606), and still the difference was nonsignificant.

### Sensitivity Analyses

Overall, there was not an individual trial influencing the overall effect estimates significantly. After removing each trial and calculating the overall estimates for the remaining trials, the significance of the WMD or OR remained materially unchanged (data not shown).

### Meta-Regression Analyses

A set of meta-regression analyses were conducted accordingly to explore the extent to which trial-level variables account for heterogeneity among the effect estimates. Unfortunately, none of the examined trial-level confounders contributed to the changes of effect estimates between the robotic ablation and manual ablation approaches (data not shown). It is widely accepted that meta-regression analysis, albeit enabling continuous variables to be considered, does not have the methodological rigor of a properly designed study that is intended to test the effect of these covariates formally.

## Discussion

To the authors' knowledge, this is to date the first meta-analysis synthesizing data on the comparison of robotic ablation with manual ablation based on 8 clinical articles and 792 patients for the treatment of atrial fibrillation. The principal finding of this study was the greater reductions of both fluoroscopic time and dose-area product in patients with robotic ablation than with manual ablation, especially in randomized clinical trials. However, caution is urged about the interpretation of these comparisons due to the evident heterogeneity. Moreover, although the success rate of catheter ablation was relatively high by using robotic ablation, significance was not reached likely due to the lack of statistical power or the initial learning stage of this novel technique.

The application of robotic ablation in clinical routine is still in its infancy, and the benefits of this novel technique for catheter ablation in treating atrial fibrillation are unquestionable [16], including excellent catheter stability and accuracy of its movement, reduced fluoroscopic time, catheter contact monitoring, improved comfort of the operator during the procedure as they can sit most of the time unexposed to radiation and a very short learning curve potentially allowing for more complicated procedures [17]. Various attempts to summarize the existing evidence have been made in recent years, but always in the context of a narrative review of the literature [17]-[19]. This therefore prompts us to quantitatively assess the superiority of the robotic ablation over the manual ablation in the form of a meta-analysis. Despite approximate 9 minutes in fluoroscopic time were averagely saved in patients with robotic ablation relative to with manual ablation in our findings, total procedure duration remained similar between the two procedures, which is likely attributable to the longer patient prepping time for robotic ablation. This is also understandable because the findings of most enrolled trials were based on the initial experience of robotic ablation systems. More importantly, shorter fluoroscopic time to scattered x-ray is beneficial not only to the operator's health during a long interventional career and but also to the patients themselves [20], as partly reflected by the reduced dose-area product in our overall analyses. However, this potential benefit might be balanced out by the high costs, increasing the burden of patients. Nevertheless, we believe that with the accumulation of practical evidence, procedures will be greatly improved by using the robotic ablation systems.

However, a note of caution should be added because since heterogeneity in our principle findings might potentially limit the interpretation of the pooled effect estimates. Of note, study design might be a potential source of heterogeneity between trials in the subgroup analyses of this meta-analysis because heterogeneity between trials totally disappeared for fluoroscopic time after restricting analysis to randomized clinical trials. To further account for the contribution of examined trial-level continuous moderators to the overall heterogeneity, we undertook a set of meta-regression analyses, but unfortunately we failed to tease out any contributory factors. It should be noted, however, this meta-regression analysis involves trials of limited sample size, rendering it underpowered to detect a small or moderate effect, and definitively there is a need for further large trials.

Despite the clear strengths of this meta-analysis including the low probability of publication bias, and the robustness of statistical analyses, interpretation of our findings, however, should be viewed in light of several limitations. First, six of ten qualified trials were performed on a nonrandomized design, raising the potential existence of potential biases. On the other hand, although randomized trials can minimize bias and are regarded as the gold standard for quantifying effect estimates, they may not be reflective of patients treated in general clinical practice [21]. Second, there was strong evidence of heterogeneity in a majority of our overall and subgroup analyses, limiting the interpretation of pooled effect estimates. Third, the total sample size of this meta-analysis was not large enough to draw a firm conclusion, such that our findings need to be validated in a large, well-designed clinical trial, and fortunately the ongoing prospective international man-and-machine trial by Rillig et al is designed to fully address the superiority of robotic ablation over manual ablation [22]. Fourth, the fact that study patients were all Caucasians limited the generalizability of our findings, necessitating the future validation in other ethnics. Last but not the least, as with all meta-analyses, despite the low probability of publication bias reported in this meta-analysis, selection bias cannot be completely excluded, since we merely identified articles from the English journals and published trials.

In summary, this study confirmed and extended previous observations by quantifying the great reductions of fluoroscopic time and dose-area product in patients with robotic ablation than with manual ablation, especially in randomized clinical trials. For practical reasons, with the accumulation of data from large randomized clinical trials, successful validation of our findings will revolutionize the current clinical practice and healthcare system by bringing great benefits to doctors and patients alike in the near future.

## Supporting Information

Checklist S1 **The PRISMA checklist.**
(DOC)

## References

[1] Verma A, Sanders P, Macle L, Deisenhofer I, Morillo CA, et al.. (2012) Substrate and Trigger Ablation for Reduction of Atrial Fibrillation Trial-Part II (STAR AF II): design and rationale. Am Heart J 164: 1-6 e6. 10.1016/j.ahj.2012.04.002 22795275

[2] Steven D, Servatius H, Rostock T, Hoffmann B, Drewitz I, et al (2010) Reduced fluoroscopy during atrial fibrillation ablation: benefits of robotic guided navigation. J Cardiovasc Electrophysiol 21: 6–12. 19793149 10.1111/j.1540-8167.2009.01592.x

[3] Nolker G, Gutleben KJ, Muntean B, Vogt J, Horstkotte D, et al (2012) Novel robotic catheter manipulation system integrated with remote magnetic navigation for fully remote ablation of atrial tachyarrhythmias: a two-centre evaluation. Europace 14: 1715–1718. 22719063 10.1093/europace/eus169

[4] Smilowitz NR, Weisz G (2012) Robotic-assisted angioplasty: current status and future possibilities. Curr Cardiol Rep 14: 642–646. 22833302 10.1007/s11886-012-0300-z

[5] Thomas D, Scholz EP, Schweizer PA, Katus HA, Becker R (2012) Initial experience with robotic navigation for catheter ablation of paroxysmal and persistent atrial fibrillation. J Electrocardiol 45: 95–101. 21714971 10.1016/j.jelectrocard.2011.05.005

[6] Rillig A, Meyerfeldt U, Tilz RR, Talazko J, Arya A, et al (2012) Incidence and long-term follow-up of silent cerebral lesions after pulmonary vein isolation using a remote robotic navigation system as compared with manual ablation. Circ Arrhythm Electrophysiol 5: 15–21. 22247481 10.1161/CIRCEP.111.967497

[7] Dagres N, Varounis C, Flevari P, Piorkowski C, Bode K, et al (2009) Mortality after catheter ablation for atrial fibrillation compared with antiarrhythmic drug therapy. A meta-analysis of randomized trials. Am Heart J 158: 15–20. 19540387 10.1016/j.ahj.2009.05.012

[8] Moher D, Liberati A, Tetzlaff J, Altman DG (2009) Preferred reporting items for systematic reviews and meta-analyses: the PRISMA statement. Ann Intern Med 151: 264-269, W264. 10.7326/0003-4819-151-4-200908180-00135 19622511

[9] DerSimonian R, Kacker R (2007) Random-effects model for meta-analysis of clinical trials: an update. Contemp Clin Trials 28: 105–114. 16807131 10.1016/j.cct.2006.04.004

[10] Bowden J, Tierney JF, Copas AJ, Burdett S (2011) Quantifying, displaying and accounting for heterogeneity in the meta-analysis of RCTs using standard and generalised Q statistics. BMC Med Res Methodol 11: 41. 21473747 10.1186/1471-2288-11-41 PMC3102034

[11] Malcolme-Lawes LC, Lim PB, Koa-Wing M, Whinnett ZI, Jamil-Copley S, et al (2013) Robotic assistance and general anaesthesia improve catheter stability and increase signal attenuation during atrial fibrillation ablation. Europace 15: 41–47. 22915788 10.1093/europace/eus244

[12] Duncan ER, Finlay M, Page SP, Hunter R, Goromonzi F, et al (2012) Improved electrogram attenuation during ablation of paroxysmal atrial fibrillation with the Hansen robotic system. Pacing Clin Electrophysiol 35: 730–738. 22494451 10.1111/j.1540-8159.2012.03381.x

[13] Tilz RR, Chun KR, Metzner A, Burchard A, Wissner E, et al (2010) Unexpected high incidence of esophageal injury following pulmonary vein isolation using robotic navigation. J Cardiovasc Electrophysiol 21: 853–858. 20233267 10.1111/j.1540-8167.2010.01742.x

[14] Kautzner J, Peichl P, Cihak R, Wichterle D, Mlcochova H (2009) Early experience with robotic navigation for catheter ablation of paroxysmal atrial fibrillation. Pacing Clin Electrophysiol 32 Suppl 1S163–166. 19250085 10.1111/j.1540-8159.2008.02277.x

[15] Di Biase L, Wang Y, Horton R, Gallinghouse GJ, Mohanty P, et al (2009) Ablation of atrial fibrillation utilizing robotic catheter navigation in comparison to manual navigation and ablation: single-center experience. J Cardiovasc Electrophysiol 20: 1328–1335. 19656244 10.1111/j.1540-8167.2009.01570.x

[16] Willems S, Steven D, Servatius H, Hoffmann BA, Drewitz I, et al (2010) Persistence of pulmonary vein isolation after robotic remote-navigated ablation for atrial fibrillation and its relation to clinical outcome. J Cardiovasc Electrophysiol 21: 1079–1084. 20455974 10.1111/j.1540-8167.2010.01773.x

[17] Jan P, Jan Š (2012) Robot-assisted navigation in atrial fibrillation ablation—Of any benefits? Cor et Vasa 54: e408–413.

[18] Nazarian S (2010) New technologies and therapies for cardiac arrhythmias. Minerva Cardioangiol 58: 731–740. 21135812

[19] Bai R, L DIB, Valderrabano M, Lorgat F, Mlcochova H, et al (2012) Worldwide experience with the robotic navigation system in catheter ablation of atrial fibrillation: methodology, efficacy and safety. J Cardiovasc Electrophysiol 23: 820–826. 22509886 10.1111/j.1540-8167.2012.02316.x

[20] Picano E, Vano E (2011) The radiation issue in cardiology: the time for action is now. Cardiovasc Ultrasound 9: 35. 22104562 10.1186/1476-7120-9-35 PMC3256101

[21] Piccini JP, Berger JS, O′Connor CM (2009) Amiodarone for the prevention of sudden cardiac death: a meta-analysis of randomized controlled trials. Eur Heart J 30: 1245–1253. 19336434 10.1093/eurheartj/ehp100

[22] Rillig A, Schmidt B, Steven D, Meyerfeldt U, L DIB, et al (2013) Study design of the man and machine trial: a prospective international controlled noninferiority trial comparing manual with robotic catheter ablation for treatment of atrial fibrillation. J Cardiovasc Electrophysiol 24: 40–46. 23131063 10.1111/j.1540-8167.2012.02418.x

